# Retraction Note: Combined laser-activated SVF and PRP remodeled spinal sclerosis via activation of Olig-2, MBP, and neurotrophic factors and inhibition of BAX and GFAP

**DOI:** 10.1038/s41598-025-28701-3

**Published:** 2025-11-19

**Authors:** Mariam F. Farid, Noha A. E. Yasin, Asmaa K. Al-Mokaddem, Marwa A. Ibrahim, Yara S. Abouelela, Hamdy Rizk

**Affiliations:** 1 Department of Anatomy and Embryology, Faculty of Veterinary Medicine, Cairo University, Giza Square, Giza, 12211 Egypt; 2 Department of Cytology and Histology, Faculty of Veterinary Medicine, Cairo University, Giza, 12211 Egypt; 3 Department of Pathology, Faculty of Veterinary Medicine, Cairo University, Giza, 12211 Egypt; 4 Department of Biochemistry and Molecular Biology, Faculty of Veterinary Medicine, Cairo University, Giza Square, Giza, 12211 Egypt

Retraction of: *Scientific Reports* 10.1038/s41598-024-52962-z, published online 07 February 2024

The Editors have retracted this Article.

After publication, concerns were raised regarding several images. Figure 4 control positive group, panels T1, T2 and Axial T2 were found to overlap with Figure 5 EB-treated group, panels T1, T2 and Axial T2 in^1^, depicting different treatments. Figure 5 panels A and B appear to overlap with a different colour gradient in Figure 3 EMSCs panels B and C in^2^. Furthermore, in Figure 8, panels a, d and e, and panels k n and o appear to overlap at different magnifications. The Authors stated this was intentional to illustrate how the cells were organised. However, this was not indicated in the figure legend in the same way it was declared in other instances. The Authors were able to submit some original images upon request, but this was not sufficient to resolve all of the concerns raised. The Editors have lost confidence in the data and conclusions of this Article.

Hamdy Rizk, Noha Yassin, Mariam Fayez and Yara Sayed disagree with this retraction. All other Authors have not responded to correspondence from the Publisher regarding this retraction.
